# The Dysenteries

**Published:** 1919-04-19

**Authors:** 


### THE DYSENTERIES.

### A Review of Recent Experience.

At the recent B.M.A. meeting, although necessarily condensed and abbreviated by the shortness of time allotted to each subject, the opening papers and discussion on these diseases gave to the medical world an extremely useful *précis* of both the present state of our knowledge and the requirements for the immediate detection of instances of these infections. Coming as newly notifiable and, in future, possibly occurring conditions in Great Britain it is of the greatest importance that the essentials of their diagnosis, investigation and prevention should be widely known, and leaving aside the detailed laboratory work, the exact differentiation of pathogenic strains of organisms and the finer points upon which the pathologists find it necessary to enlarge we will attempt to extract those immediate considerations which will face the practitioner who encounters cases or minor outbreaks which the past years of war threaten to leave in their wake.

### Carriers of Bacillary Dysentery.

As Colonel Dudgeon pointed out in the first paper, bacillary dysentery is now viewed as an infectious disease due to a specific set of organisms, and characterised by fever, tenesmus, diarrhœa, followed by the passage of blood and mucus, but although this is the typical clinical picture, it must be realised that dysentery bacilli may be isolated from people who have had diarrhœa for a period of 24 hours only, without any other clinical manifestation. The importance of such cases lies not only in the apparent discrepancies they may produce in epidemiological and serological study of the infection, but also in the difficulty they cause in the complete detection of carriers among civilians and troops returning to Great Britain and the Colonies. Therefore must the public health departments and practitioners be prepared with the readiest methods and technique immediately to remedy the effects of such invasion.

In the first place they must remember that, if any reliance is to be placed in a negative finding obtained from an otherwise suspicious case, the samples of stools *must* be received in the laboratory in a fresh state and there dealt with immediately. If the specimen consist of blood and mucus alone, then a few hours' interval is permissible, but if fæces are present it is absolutely essential to avoid delay. The bacilli flourish in an alkaline, but suffer lowering of vitality and rapid death in an acid medium. Blood and mucus stools are alkaline when passed, but if fæces be present and the specimen allowed to stand any length of time at ordinary temperature strong acid reaction will gradually develop. Experimental evidence has shown that the addition of three per cent. normal sodium hydrate to the specimen will largely counteract this effect and should be generally adopted. Attention to this apparently small yet essential point in sample collecting considerably improves the certainty of a laboratory finding.

Dr. E. H. Lepper emphasises the importance of examination of stool specimens for mucus threads. If there is a known history of previous infection, mucus implies persistence of the organism and also it is only in these mucoid patches that the bacilli are to be found. The greatest care should be taken, therefore, with any particular case to choose only those specimens which contain mucus and not to allow the sample to be taken in a routine or haphazard way by an inexperienced attendant. Results show that during convalescence about six per cent. of the patients still harbour the bacilli, while after three months the figure falls to 1.5 per cent., but after long periods there are still an appreciable number of carriers apart from the totally unrecognised cases mentioned earlier. Therefore this plea for the most careful attention to essential details of collection, and the need becomes more obvious when we learn from Colonel Dudgeon that many apparently completely recovered men still give a positive agglutination test, analogous to the Widal reaction for typhoid, against one or other of the specific dysenteric sera.

### Conveyance of Infection.

The two main channels coming under consideration with the bacillary disease are water and flies. In the instance of the former, the recent conditions of war in the East provided experience of the germ in all varieties of water, natural and artificial, but upon its vitality under such conditions, observations must be pressed further before absolute preventive measures can be assured. Investigational work was at that time greatly hampered by circumstances, but a few facts stand out definitely in the conclusions drawn, and we know that dysentery bacilli were cultivated up to a period of 576 hours from river water stored at ordinary temperatures, and up to 336 hours from infected sterilized water under like conditions. Also, the bacillus, when placed in previously chlorinated water could live for many hours. Therefore, although in every case storage at a temperature to 37° C. greatly reduced the period of the germ's life, yet under normal conditions all water is both able and likely to provide a ready means of transference, particularly as the bacillus retains its cultural, serological, and a great extent of its pathogenic power under these conditions.

Next to consider the most ready indirect infecting agent—the fly: two years' experience on the Macedonian front showed that while dysentery occurred to some extent among the troops throughout all the warmer months, the two periods when it assumed its greatest prevalence—namely, spring to early summer, and late summer to early autumn—were the times when the fly pest was at its worst. In experiments by Capt. J. F. Taylor, flies made to feed on material known to contain Shiga or Flexner bacilli were allowed to contaminate surfaces, (1) by walking; (2) with their fæces; (3) from the legs; and it was found that the fæces most frequently showed the presence of the organisms but that it

**The Dysenteries**—(*continued*).

was difficult to recover the germs from the insects by any means after 24 hours. In a few cases, dysentery bacilli were isolated from flies caught in the wards, latrines, etc., of hospitals and though the positive proportion was only 1 in 1,500 captured, when one considers the short period of infectivity mentioned above, the combined facts strongly suggest the prominent part played by flies in dissemination of the disease. There are many finer points to be worked out, but we can assume the infection to be, as well as by direct contamination, both fly and water-borne, and it now remains to find the weak points in the formidable combination of attack and develop our counter-measures accordingly.

### Amœbic Dysentery.

In the second opening paper, Dr. Warrington Yorke limited his remarks to three aspects of amœbic dysentery, diagnosis, the importance of "carriers," and the chances of the disease becoming endemic in this country. The routine attempt to detect carriers of *entamœba histolytica* among soldiers invalided to this country was undertaken for two main reasons: (1) carriers were regarded as dangerous to the community; (2) so long as a man harboured the cysts it was considered that he might be subject to a relapse of acute dysentery or to hepatic abcess. Not only were 11.5 per cent. of convalescents found to remain infected, but also a similar proportion of men who were invalided for diseases other than dysentery showed the cysts in their stools, but even more striking was the fact that a considerable number of positive results were obtained from troops that had never been out of England. Following this line of investigation, it was found that many of these infections could be traced back through whole families, in which it was most unlikely that the original amœbæ came from a present war zone, and also that the organism was markedly present among certain communities such as the miners. Figures by Wenyon and O'Connor on the prevalence of the amœbæ in Egypt, among both white and native carriers, correspond to a marked degree with the figures obtained in this country, and it appears possible that there has for years been a transference of these organisms to and from both this country and the East. War conditions have intensified the process, and the truth of early predictions that the infection was unlikely to gain a footing in this country owing to its relatively good sanitary conditions appears to depend upon the question of whether the introduction of the disease to England is old-standing or really of recent occurrence. Before the war undoubtedly the opportunity for importation existed, and also, in many quarters, the necessary conditions for spread. At the same time, no cases of acute amœbic dysentery have yet been recorded here, and this suggests that some additional factor, at present unknown, is necessary before acute symptoms develop. It is interesting to note Walker and Sellards' classical experiments when twenty native volunteers were fed upon infected material. Ninety per cent. of these people became parasitized, but only 20 per cent. developed amœbic dysentery, with an incubation period varying from twenty to ninety-five days.

### Diagnosis of Sub-Acute Disease.

It is probable that owing to attention having, in this country, been so largely focussed on the "carrier" problem, the presence of actual dysenterics has been to some extent overlooked. The chief difficulty arises with sub-acute dysenteric. Cysts are readily recognised outside the body, and they have considerable vitality, but in its vegetative stage the parasite quickly becomes unrecognisable. In no other clinical laboratory investigation is more experience needed and closer touch between the medical officer and the bacteriologist necessary. The disadvantage in delay was emphasised in the instance of the bacillary disease. Here it is even more important. Attention is also drawn to the still present difficulty in distinguishing the *vegetative* stages of *E. hystolitica* and *E. coli*. Dr. Yorke's own view is that if entamœbæ are found in numbers in stools of a person with sub-acute symptoms the case should be regarded as amœbic dysentery.

### Treatment of Sub-Acute Cases.

The lecturer gave, in conclusion, a method of treating these and relapsing cases which he found invariably cleared the stools of amœbæ—a result which cannot be claimed for emetine alone—and speedily caused the disappearance of acute symptoms.

A preliminary saline purge is given unless acute symptoms have persisted several days, in which case it is unnecessary.

Emetine hydrochloride gr. i. is injected subcutaneously, and bismuth subnitrite ℥ii. or iii., suspended in milk or water, is given by the mouth three or four times daily for twelve days.

Occasionally a morning saline may be necessary if the bismuth causes constipation.

### The General Discussion.

Many points of both clinical and technical interest were raised in the general discussion, and we select a few of those bearing upon the future of the disease in this country.

Sir William Osler said that he himself was more interested in amœbic than in bacillary dysentery, because he believed that he had stood sponsor to the English-speaking world for the pathogenic respectability of the amœba. But it was interesting to note how different had been the amœbic dysenteries seen during the war from the amœbic dysenteries which had been observed in such numbers shortly after the opening of the Johns Hopkins Hospital, in respect particularly to the more serious complications, which in these later cases were relatively more rare. In the former instance they had 25 per cent. of the cases with hepatic abscess, but cases of hepatic abscess in this country—among the dysenterics from the East and from France—had been comparatively rare, and moreover, the cases had not the same intensity, and the mortality had not been nearly so great. No doubt that had been due to the introduction of the emetine treatment. But the point of practical interest was whether they were likely to have an increase in the number of amœbic dysentery cases in this country. He did not

**The Dysenteries**—*(continued)*.

think there was any chance of it. They had had nearly 40,000 patients in the base hospital at Oxford—a large proportion of them from France and from the East—and there had been no single case of amœbic dysentery admitted to the infirmary from the civilian population during the war. If, as was stated, 5 or 6 per cent. of the indigenous population harboured the amœba, let them continue to harbour it; it certainly had not done them any harm. He had himself seen but one definitely indigenous case in this country.

Lt.-Col. Duncan Graham thought a microscopical examination specially important in the large percentage of cases coming into the hospital diagnosed as diarrhœa. The spread of dysentery came from undiagnosed or uncured cases of bacillary dysentery, and these should not be called carriers, but should be recognised definitely for what they were.

Dr. O'Connor spoke of his experience in Egypt, where amœbic dysentery was known to be exceedingly prevalent long before the war. During 1916-17 he examined 1680 Egyptians who were associated with our army on the Sinai peninsula, and he found the carrier incidence very much higher than and out of all proportion to that amongst the British troops. As to the sanitary conditions of this country being conducive to the spread of disease, while in certain cases the sanitation was certainly very defective, there was never a fly plague at all equivalent to that which was found in Egypt and other tropical countries. He thought, on the whole, that the sanitary conditions here did not conduce to the spread of amœbic dysentery.

Major Adrian Stokes said that the first epidemic of dysentery on the Western front was in 1916; it did not occur until the German trenches had been taken, and it was apparently acquired from the Germans. From the army point of view he believed that the only way of driving out dysentery was by some form of prophylaxis.

Sir William Leishmann said that as to the diagnosis of bacillary dysentery there were three alternatives: clinical diagnosis, bacteriological diagnosis (which was the ideal), and microscopical examination of stools. He had come more and more to think that the last-named was the best they could do. He referred appreciatively to the work of the Dysentery Committee.

